# Carl Flügge, one of the last holistic hygienists and discoverer of droplet transmission of infectious diseases

**DOI:** 10.1007/s00430-024-00801-3

**Published:** 2024-08-02

**Authors:** Peter R. Donald, Stefan H.E. Kaufmann, Dagmar Schaub, Stephanie Thee, Christoph Lange

**Affiliations:** 1 Desmond Tutu TB Centre, Department of Paediatrics and Child Health, Faculty of Medicine and Health Sciences, University of Stellenbosch, Tygerberg, Western Cape Province South Africa; 2 Max Planck Institute for Infection Biology, Berlin, Germany; 3 Max Planck Institute for Multidisciplinary Sciences, Göttingen, Germany; 4 Hagler Institute for Advanced Study, Texas A&M University, College Station, USA; 5 Charité – Universitätsmedizin Berlin, Corporate Member of Freie Universität Berlin and Humboldt- Universität zu Berlin, Berlin, Germany; 6 German Center for Infection Research (DZIF), Partner Site Hamburg- Lübeck-Borstel-Riems, Lübeck, Germany; 7 Department of Clinical Infectious Diseases, Research Center Borstel, Leibniz Lung Center, Borstel, Germany; 8 Department of Pediatric Respiratory Medicine, Immunology and Critical Care Medicine and Cystic Fibrosis Center, Charité – Universitätsmedizin Berlin, Corporate Member of Freie, Universität Berlin and Humboldt-Universität zu Berlin, Berlin, Germany; 9 Respiratory Medicine & International Health, University of Lübeck, Lübeck, Germany; 10 Baylor College of Medicine and Texas Childrens’ Hospital, Houston, TX USA

**Keywords:** Carl Flügge, Tuberculosis, Droplet transmission, Hygiene

## Abstract

Carl Flügge is best known for the promotion of studies demonstrating the transmission of all manner of infections, but particularly tuberculosis, by coughed droplets. But it is seldom recognised that Flügge was also influential in a number of other fields comprising the practice of hygiene. One-hundred years following his death in 1923, we review literature related to the studies of Flügge and his colleagues and students and illustrate the particular emphasis he laid upon the environment within which disease and its transmission might be fostered or prevented, embracing and studying aspects essential to the health of any community ranging from fundamental microbiology in the laboratory to subjects as disparate as housing, clean water supply, nutrition, sanitation, socio-economic circumstances and climate. Very early in his career he promoted breast feeding for the prevention of seasonal gastro-enteritis and later the sheltering of cough as a means of preventing the transmission of infected respiratory droplets, not only as regards tuberculosis, but also concerning all manner of other respiratory infections. By the time of Flügge’s death the complexification of available scientific methodologies comprising hygiene made it difficult for any individual to comprehend and study the wide range of hygiene-related subjects such as Flügge did. Carl Flügge was one of the last holistic hygienists and an originator of the study of environmental health as a pillar of hygiene.

## Introduction

Carl Flügge is best known for studies demonstrating potential infection transmission by aerosolized, coughed droplets, but in particular regarding tuberculosis (TB) transmission [1–4]. During the recent COVID-19 pandemic a number of reviews related to potential aerogenous transmission of SARS-CoV-2 appeared citing Flügge’s studies. During a search in November 2023 of PubMedCentral under the heading “COVID-19 transmission and Carl Flügge” 26 references were recorded. While a minority of papers provided a nuanced assessment of Flügge’s achievements [5–7], in a majority reference to Flügge’s research was a brief perfunctory note that Flügge had demonstrated that coughed droplets could be a vehicle for infection transmission; Flügge’s studies, however, were more complex and far reaching than is usually appreciated. Further Flügge’s studies and those of his students, colleagues and co-workers were influential in many other aspects of health care and other fields. The 100th anniversary of Flügge’s death in 2023 is an appropriate time to review the career of a remarkable scientist, microbiologist, physician, epidemiologist and hygienist in the fullest sense of the word. Flügge himself defined hygiene as *“That part of medical science that concerns itself with the environment surrounding people and studies those factors that frequently and to a considerable extent disturb the capability of the human organism to function optimally”* [4].

Against the above background we present a narrative review of Carl Flügge’s wide-ranging career drawing on back-searching of Flügge’s publications and those of his collaborators, his colleagues and contemporaries and personal literature collections. In particular we emphasize his collaborators’ studies of *Mycobacterium tuberculosis* (*Mtb*) transmission noting neglected features of Flügge’s studies and in particular his persistent concern for all aspects of the environment in which infection transmission occurs and the environmental conditions that predispose to all manner of human disease. Throughout this review we use the term droplet, but note that Flügge knew that considerable drying might occur in the smallest coughed droplets but preferred the term droplet to emphasize the initial water content of the coughed material in contrast to the structure of dried dust particles at the time of aerosolization [8].

### Origins and early career

Carl Flügge’s research activities are briefly summarised in Table 1. Flügge was born into a family of physicians on 12th September 1847 in Hanover and from age 18-years studied medicine in Göttingen where his lecturers included Fredrich Wӧhler, Jacob Henle and Wilhelm Weber; amongst contemporary students was Robert Koch (1862–1866). Assessing the careers of both Flügge and Koch it is note-worthy that Henle in 1840 published a paper dealing with the concept of miasma and infectious diseases “*Von den Miasmen und Contagien und von den miasmatisch-contagiösen Krankheiten*”. (Concerning miasma and contagion and miasmatic-contagious diseases) [9]. In this article Henle supported the then unpopular microbial contagion theory of the Renaissance forerunner of modern epidemiology, Girolamo Fracastoro, who stated “The material of contagions is not only an organic but a living one and is indeed endowed with a life of its own, which is, in relation to the diseased body, a parasitic organism” [10]. The careers of both Flügge and Koch were likely influenced by Henle’s ideas.

**Table 1:** Significant contributions by Carl Flügge to the practice of Hygiene

Year	Topic	Contributions by Carl Flügge
1881–1887	Hygiene: Microbiology and Medicinal Chemistry	Teaching in Göttingen of first formal university courses in Hygiene and Medicinal Chemistry. Identification of the cause of tetanus. Publication of “Die Mikroorganismen”. In 1883 became the first professor in Hygiene and Medicinal Chemistry in Prussia [11, 12].
1885	Founding of Zeitschr f Hygiene	Together with Robert Koch the founding and first publication of Zeitschrift für Hygiene; title at present Medical Microbiology and Immunology.
1893	Cholera	An account on the mechanisms of the spread of cholera and their importance for the containment of the disease [13].
1897	Identification of airborne transmission of infectious disease by droplets and dust.	Demonstration that droplets expelled during coughing, sneezing, breathing or speaking can transmit microorganisms. This finding laid the groundwork for understanding the spread of respiratory infections and the development of infection control measures. Dust particles infected with a microorganism can also become airborne in the presence of sufficiently strong air currents and complete drying of sputum particles [1, 3].
1898	Formaldehyde introduced for disinfection of potentially infected buildings or utensils using the "Breslau Apparatus for its production	Demonstration of the disinfection properties of formaldehyde used in contaminated living quarters or hospital wards and also for the disinfection of various utensils and equipment [4, 14].
1912	Identification of the association between infant deaths and summer increases in gastro-enteritis and preventive value of breast feeding	Data presented identifying a much higher infant mortality amongst infants fed cows’ milk in comparison to those breast fed. Infant mortality was also associated with higher summer temperatures and the occurrence of gastro-enteritis that was more common amongst the poorer social classes in Berlin. A simple methodology developed for sterilization of cow’s milk for infant feeding [4].
1916	Assessed and criticized uncontrolled urban development and its health consequences	Description and evaluation of the relationship of health and disease in relation to the environment and in particular to the urban environment prevailing in German cities during his career [15].

When Flügge studied medicine, Germany’s major cities faced serious challenges resulting from rapid urbanization of considerable numbers of people requiring housing, clean water and refuse and sewage removal; rampant infectious diseases caused epidemics with loss of life; outbreaks of cholera focused attention on the necessity of attending to these problems. Rudolf Virchow emphasized the role of poor housing, crowded hospitals, prisons, schools and factories in spreading infectious diseases and development of malnutrition [16]. Fortuitously the rise of these problems was matched by development of laboratory sciences encompassing chemistry, microbiology and pharmacology; these were drawn together with sociology and epidemiology under the all-embracing banner of hygiene. In 1865 Max Pettenkofer, known for advocating sanitation for disease prevention and favouring the miasma theory, became professor of hygiene in the first Hygiene Institute in Germany at Munich University [17].

Following military service Flügge gained experience in a range of medical and hygiene institutions in Germany being exposed to many scholars prominent in the disciplines comprising hygiene. Between 1875 and 1878 he studied experimental hygiene in Leipzig with Franz Hoffmann, himself a student of Max Pettenkofer under whom Flügge later spent a semester in Munich. Significantly, considering Flügge’s later interests, Franz Tappeiner was then in Munich conducting early studies of aerogenous transmission of TB with nebulised TB sputum [18]. Also in Munich Hans Buchner experimented with aerosolization of infectious organisms from fluids, developing instruments for aerosolization of infectious fluids [19, 20].

In 1878 after qualifying as state physician Flügge gave courses at a private laboratory in the methodology of hygiene investigations, maintained a medical practice in Schӧneberg near Berlin (now a district of Berlin) and wrote “*Beiträge zur Hygiene*” (A Contribution to Hygiene) [21]. In 1881 the first edition of “*Lehrbuch der hygienischen Untersuchungsmethoden*” (Text book of Hygiene Investigations) appeared and became a widely praised standard hygiene textbook [22].

## Called to Göttingen

From 1881 to 1887 Flügge returned to Gӧttingen working in the Physiology Institute under George Meissner, lecturing in medicinal chemistry and developing the first Prussian Institute for Hygiene and Medicinal Chemistry. He became ordinarius promoting hygiene and microbiology as independent subjects and identified several new pathogenic organisms including the tetanus bacillus assisted by his student Nicolaier [23]. Early 1881 Robert Koch visited Flügge in Gӧttingen and they developed a mutually fruitful relationship, discussing modern microbiological techniques while Flügge provided Koch with manuscripts, tables and advice regarding hygiene lectures, drawing Koch’s attention to hygiene as a wide-ranging academic subject [24]. In 1885 Flügge and Koch established the influential journal “Zeitschrift für Hygiene” (Journal of Hygiene, now Medical Microbiology and Immunology)) and Flügge’s text-book “*Die Mikroorganismen*” (The Microorganisms) published in 1886 assisted the early standardization of microbiological techniques [12].

## Called to Breslau

**Fig. 1:**
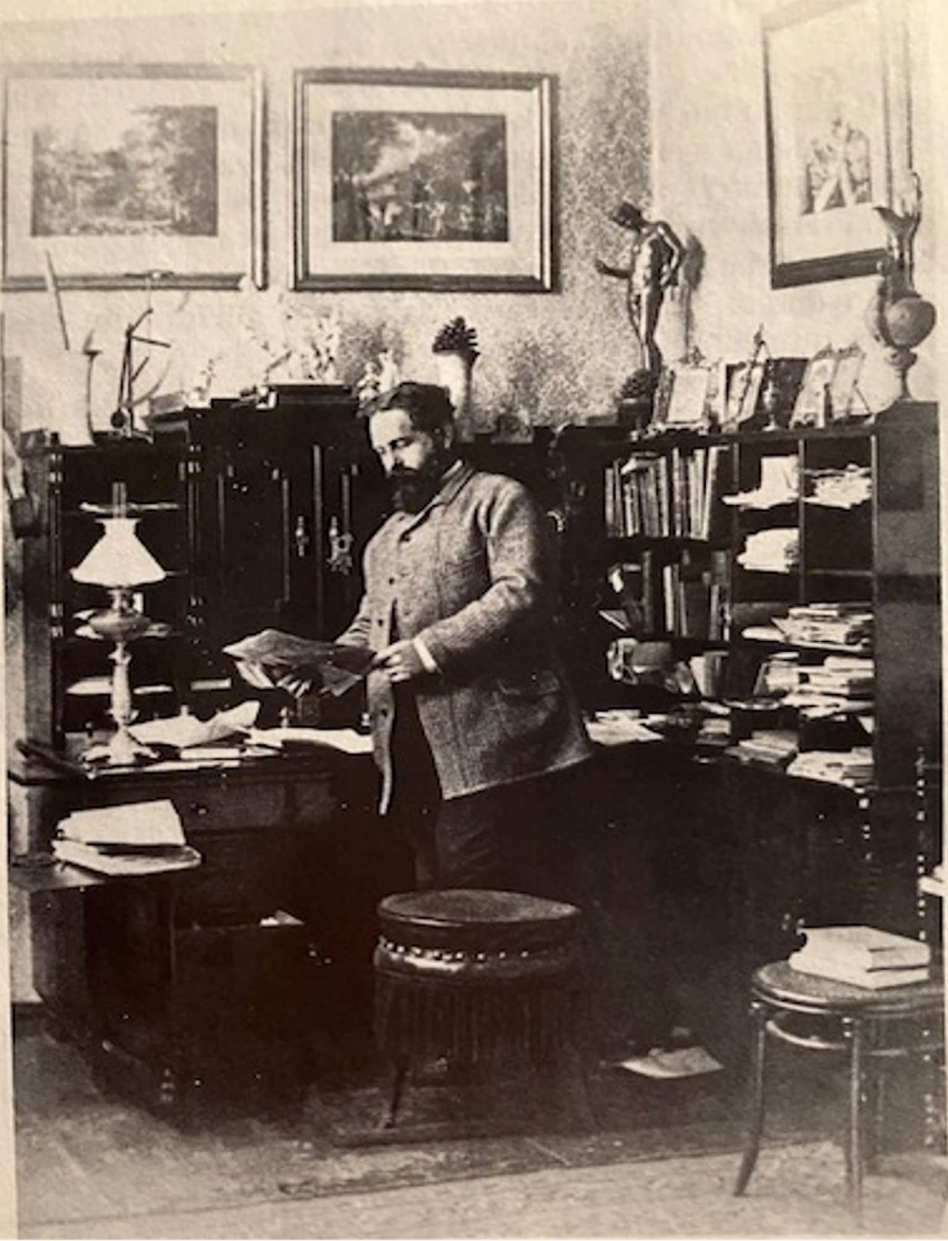
Carl Flügge in his office in Breslau approximately 1900 [27]

In 1887 Flügge responded to a call to Breslau (Wroclaw) entering arguably his most productive period. During the next 22-years Flügge developed a research institute devoted to studying infectious diseases and their transmission. He gathered a team of younger researchers who, under his direction, carried out interlocking investigations focussed on infectious diseases and their transmission, laying a foundation stone of our knowledge of aerogenous infection transmission (Fig. 1).

On Flügge’s arrival in Breslau the Hygiene Institute was housed in temporary accommodation, but new buildings, planned with Flügge’s assistance, were begun in 1897 and completed two years later. These comprised the Hygiene Institute, the Physiology Institute, a Pharmacology Institute and Clinic and numerous laboratories, a lecture hall and a library. Particularly noteworthy were the rabies and climatology stations [25].

Flügge’s 1897 article “*Ueber Luftinfection*” (Concerning aerogenous Infection) became his most frequently referenced work where he summarized conclusions regarding the existing literature and studies conducted under his direction in Breslau [1]. He collated evidence that a variety of infections can be transported following aerosolization by coughing or even just breathing or speaking or following aerosolization of infectious agents by air currents of varying speed from various surfaces.

Although TB studies carried out by Flügge and his assistants in Breslau have drawn the most attention it is notable that they comprise only approximately 40% of his academic publications during his 21-years in Breslau [26]. Other subjects studied included the dynamics of infectious diseases such as diphtheria, typhus, cholera and rabies; he was active in areas regarding health administration such as water hygiene, sterilisation of rooms or buildings and the overall living conditions of poorer sections of the population [25].

### TB and other infectious disease studies in Breslau undertaken by Flügge and his colleagues

Robert Koch in his 1884 review of his TB research stated that there was no doubt that TB was a respiratory infection and that coughing by pulmonary TB (PTB) individuals loosened highly infectious sputum particles that if aerosolized could be inhaled, but he noted that these particles were big and unlikely to remain suspended in air for long [28]. This problem seemed resolved when George Cornet also demonstrated that *Mtb*-infected small dried sputum particles and fibres from infected clothing, bedding, handkerchiefs or carpets when aerosolized transmitted *Mtb* infection to guinea pigs [29]. Cornet demonstrated TB infected dust particles were frequently present in surroundings frequented by PTB patients.

Flügge’s 1897 article challenged this view of TB infection proposing that TB infection (and other infections) - could also result from inhalation of very small airborne, coughed droplets harbouring the infectious agent [1]. Early studies focussed on generation of airborne droplets during coughing and breathing. Microscope slides were extensively used placed at distances from patients varying from 50 cm to 150 cm usually at head height in front of, to the side or behind coughing or merely breathing patients, either vertical, horizontal or at a 45o angle assisting detection of smaller droplets.

The coughed droplet clouds and droplet features on microscopy were documented, in particular the contents, structure and size of droplets, the duration in the air and the microorganisms they carried. The cone shape of the coughed cloud demonstrated by the Tyndall effect was appreciated and slides were placed across the front of the cloud to study droplets at the clouds centre and periphery. Appropriate stains and microscopy enabled study of droplet contents, their structure and material impacted on slides could be washed off and cultured [30, 31].

TB related studies by Flügge’s colleagues and assistants under his mentorship in Breslau, were published in 1908 in a single volume **“***Die Verbreitungsweise und Bekämpfung der Tuberkulose”* (The spread and containment of tuberculosis); 39 chapters dealt with investigations by Flügge’s colleagues and assistants into different aspects of TB, its spread and prevention. Highlights from these papers are briefly summarized in Table 2 [32]. Here Flügge introduced the term quantum designating the number of microorganisms necessary to establish infection anywhere or alternatively the volume of breathed air necessary to establish infection.

An unexpected and striking finding was that despite sputum microscopy and culture for *Mtb* being significantly positive, often only 40–50% or fewer PTB patients coughed droplets containing *Mtb* bacilli; examined repeatedly this percentage might increase. Results of individual patients varied considerably from day to day and early morning and winter were marked by a greater proportion of patients coughing droplets containing viable *Mtb* [30, 31, 33–35]. The great majority of droplets were found within a ½ meter of coughing patients and varied in size from 20 to 500 μm - containing varying numbers of bacilli often between 100 and 500; however, droplets furthest from the patient were usually smaller ranging in size from 20 to 30 μm and much less, containing very few bacilli. It was appreciated that the droplet diameter flat on microscope slides was at least three times greater than during flight or as hanging droplets; it was likely that these smallest droplets might remain airborne longer and be more suitable for inhalation [36]. Failing this they might settle on a variety of surfaces and after drying again become aerosolised by air currents caused by household activities.

**Table 2:** Selected highlights of TB-related studies undertaken by Flügge’s colleagues and republished in *“Die verbreitungsweise und Bekämpfung Der Tuberkulose”* (the spread and containment of tuberculosis) [32]

Authors	Summary of main findings
Neisser M. 1898 [37]	After aerosolization the smallest droplets carrying bacteria and invisible to the eye might dry to dust and settle as finest dust particles. Following settling on a smooth surface those carrying *Mtb* and staphylococci were not easily aerosolized, this requiring the application of relatively strong air currents.
Beninde M. 1899 [38]	Aerosolization of viable *Mtb* attached to sputum particles adherent to dried handkerchiefs from PTB patients’ pockets was studied. If the handkerchief carried relatively little sputum, remained unused and was carried in a pocket drying for several days particles carrying viable *Mtb* could be aerosolised, but considerable energy was required to mobilise these particles.
Heymann B. 1899 [30]	Coughed droplets on microscope slides sited within a meter of 35 coughing PTB patients contained *Mtb* bacilli on microscopy in 14 cases (40%) but more on repeated investigation. The furthest placed droplets contained less mucous and extraneous material, but only 2–3 bacilli. These smaller droplets possibly originating from the oral cavity appeared more suitable for longer aerosolization and establishing infection. Of 25 guinea pigs exposed in boxes to TB patients coughing down a short tube 6 (24%) became TB-infected as demonstrated on post-mortem.
Laschtschenko P. 1899 [34]	Nine coughing PTB patients were seated individually in a sterile glass 3.2 cubic meter case for 60–90 min; TB bacilli were cultured from 4 (44%) specimens of normal saline through which the chamber air was aspirated during their presence in the chamber. Oral fluids from 9 (45%) of 20 PTB patients contained TB bacilli during cough-free periods. After coughing TB bacilli were present in droplets on microscope slides within a meter of only 4 (19%) of 21 ambulant PTB patients. Secretions with less mucinous sputum slime appear better suited to form small aerogenous droplets. Speaking, coughing, or sneezing also generated droplets from fluids in the oral cavity that could transport microorganisms.
Moeller A. 1899 [33]	Microscope slides were placed in front of 30 PTB hospitalized patients at mouth height at 1-meter distance; in 16 of 30 (53.3%) TB bacilli containing droplets were found on microscopy. These were frequently identified early morning. Bedridden sick patients did not always cough bacilli containing droplets. Two groups of patients wore a face-mask for 10–20 h daily; TB bacilli were found in the masks of all 3 seriously ill patients but in only 5 (20%) of 25 patients not seriously ill.
Sticher R.1899 [39]	Sticher studied aerosolization of TB-infected dust particles by varying wind speeds. With decreasing wind speed aerosolization decreased as did likely TB-transmission. Successful TB-transmission to guinea pigs was achieved in a minority of cases but under unnatural conditions. Even with complete drying of particles the chances of TB-transmission were low. Earlier experiments probably failed because sputum specimens were not dry enough or the air speeds too low. Only after complete drying and intense atomization of infected material and delivery by bellows directly into the guinea pig’s mouths did Cornet elicit infection [29].
Heymann B. 1901 [31]	Heymann recorded microscopy of airborne droplets formed from sputum coughed by PTB patients. The bacilli numbers coughed varied over time in the same patients. The coughed material spread in a cone with greatest droplet numbers near the patient. Droplets were spherical or oval depending on forces applied; not all droplets carried bacilli. Of 35 patients investigated once with microscope slides 14 (40%) coughed droplets containing bacilli, half with frightening numbers of bacilli. Closed culture plates placed at ceiling height opened 30-minutes after 4 coughing patients left isolation in a previously sterile glass case were positive for *Mtb* in one patient (25%) demonstrating persistent airborne survival of *Mtb* infected droplets above head-height at least 30-minutes post-cough.
Ziesché H.1907 [35]	Ziesché undertook 62 investigations in 30 PTB patients coughing towards microscope plates for half-an-hour at a distance of 40–50 cm. Examined once their coughed droplets contained tubercle bacilli in 40%; on repeated study this increased to 80%. Ziesché described bronchial and oral droplets. Bronchial droplets were middle sized with no accompanying bacteria but always TB bacilli and if mixed with saliva stood out from their background, appeared heavier and more likely to fall rapidly to ground in direct contrast to the assertions of Koeniger [40]. Further bronchial droplets experience greater propulsive currents when coughed without stopping in the mouth. With greater quantities of saliva, production of oral droplets increases.
Findel H. 1907 [41]	Findel evaluated in young guinea pigs whether primary respiratory TB-infection followed respiratory or gastro-intestinal infection; did bacilli enter the body via the oropharynx or intestines and reach the lungs lympho-haematogenously? Respiratory infection was established in the guinea pigs after inhalation of 20–40 bacilli, but amongst 14 guinea pigs fed between 191,000-382,000 TB bacilli none developed any respiratory or other TB signs; further very high infecting doses were needed to establish a gastro-intestinal TB-infection.
Kӧhlisch 1908 [42]	Viable *Mtb* were found attached to dust particles in domestic surroundings and sites frequented by infectious PTB patients. In animal experiments it was more difficult to establish a microorganism dust-particle associated aerogenous lung infection than a coughed droplet associated infection; dust particles elicited a more severe inflammatory reaction by bronchial mucosa and cilial-defence mechanisms than sputum associated droplets; on post-mortem far fewer dust particles had penetrated into distal bronchi and bronchioli than after mucous droplet infections.

For more sophisticated studies a sterilised glass chamber was constructed 3.2 cub meters in size within which a TB patient sat having put on a sterile overcoat and over-shoes and washed hands and face with sublimate. Following coughing or only normal respiratory movements by occupants, air aspirated from the chamber above head-height at predetermined speed and volume was passed through normal saline; following centrifugation the fluid sediment was cultured. Culture media or normal saline in settle-plates that could be opened and closed from outside the chamber at specific time points, were placed at different heights and distances from coughing patients within the chamber [31, 34]. Verification of *Mtb* by culture most often followed injection of concentrated specimens into a guinea pig’s abdomen. Settle plates above head height were opened 30-minutes or longer after a patient left the chamber. Similarly, air could be drawn continuously from the chamber and passed through normal saline before or after a patient left the chamber and the centrifuged sediment subject to guinea pig culture to demonstrate the airborne presence of viable *Mtb*.

Amongst the most influential early studies were Laschtschenko’s [34], demonstrating the wide aerogenous distribution of various microorganisms following coughing, sneezing or talking with varying vigour. After a researcher took fresh cultures of *Bacillus prodigiosus* (*Serratia marscens*) which produces the red dye prodigiosine into his mouth and spoke loudly or softly in a large lecture hall or carried out other manoeuvres a wide, prolonged distribution of micro-organisms occurred. *B prodigiosus was* documented at ceiling height at distances of 9-meters or more from source; these findings elicited widespread comment and concern. A similar much quoted study was later carried out in the British Houses of Parliament by Gordon, also demonstrating a similar disturbing wide distribution of *B prodigiosus* within the debating chamber (Anonymous 1906).

Regarding *Mtb* dissemination Laschtschenko assessed 21 ambulant PTB patients, not acutely ill, but only 4 (19%) coughed *Mtb-*containing droplets seen on microscopy of slides 50–100 cm in front of the patient. In further experiments 9 coughing patients were individually seated in the sterile chamber for 60–90 min, coughing when necessary. Five Petri dishes containing normal saline were placed at head-height or in the upper-most corners of the chamber at 90–170 cm distance from each patient. In 4 (44%) instances *Mtb* was identified in the Petri dishes by guinea pig infection. In later similar studies conducted by Bruno Heymann *Mtb* cultures were also positive when closed Petri dishes at ceiling height were opened 30 min and longer after subjects left the chamber. Further proof of the airborne presence of viable *Mtb* in chamber air was provided when air aspirated from the chamber for 60–90 min in the presence of PTB patients was passed through normal saline and *Mtb* bacilli cultured from the saline through which the chamber air was drawn [31]. Laschtschenko [34] also investigated 20 PTB patients at different disease stages during cough-free periods finding *Mtb* in oral secretions of 9 (45%), often in considerable numbers; he suggested that oral secretions with less mucinous sputum slime might be better suited for coughing of smallest aerogenous droplets. The motions of speaking, coughing or sneezing also generated droplets from oral fluids that could transport microorganisms.

Following Koch’s [28] and Cornet’s studies [29] and the assumption that TB transmission usually followed inhalation of airborne, dried sputum-dust particles and attached *Mtb* bacilli derived from coughed sputum particles lying on the floor or furniture or attached to clothing or bedding of active PTB individuals, early mass preventative measures aimed to ensure that sputum was collected in bowls and bottles, hygienically disposed, leaving no opportunity for it to dry and become airborne. This was also initially Flügge’s opinion. In the 3rd edition of “Grundriss der Hygiene” (1894) Flügge describes TB transmission as due to dried TB-infected sputum particles aerosolized by vigorous sweeping of carpets or equally vigorous shaking of blankets or clothing of PTB patients [43].

However, following studies of his colleagues and the publication of Flügge’s 1897 and 1899 papers *Mtb* infection following coughing and aerosolization of Mtb infected droplets could not be doubted, although the nature, duration and extent of danger remained uncertain. Paradoxically microscopy of glass slides assessing the numbers of droplets containing TB bacilli coughed by PTB patients who were either sputum smear-positive for acid-fast bacilli on microscopy or culture-positive for *Mtb* suggested that very sick patients with thick tenacious sputum might pose less risk of infection transmission than those less sick and ambulant whose muscular strength and thin fluid sputum might more easily allow aerosolisation of smallest airborne droplets carrying bacilli [2]; during several studies of droplets coughed by PTB patients, only approximately 40% were coughing *Mtb* bacill-containing droplets during a single evaluation [30, 35]. Coughing *Mtb-*containing droplets was also more frequent early mornings and during winter. Ziesché investigated 30 PTB patients with sputum microscopy-positive for acid-fast bacilli but only 12 (40%) coughed bacilli-containing droplets; this figure was similar to Heymann’s [30] findings. More frequent evaluation might increase the percentage of patients coughing bacilli.

Regarding potential inhalation of dried *Mtb*-infected sputum particles and their aerosolization Flügge later again revised his opinion following results of investigations by his colleagues [37–39, 42]. He grudgingly conceded that dried TB sputum dust particles might occasionally establish an intraperitoneal infection if injected into a guinea pig’s abdomen and if airborne might cause respiratory TB-infection in experimental animals [3]. Viable tubercle bacilli might also be attached to dried-sputum particles adhering to clothing, blankets and carpets [29]. Heymann then investigated dust from rooms frequented by phthisis patients, hospital wards and private homes finding viable *Mtb* in 18.4%, 24.3% and 12% of sites respectively [31]. Beninde [38] studied aerosolization of viable *Mtb* attached to sputum particles adherent to dried handkerchiefs. If the handkerchief carried relatively little sputum, remained unused and carried in a pocket drying for several days particles carrying viable *Mtb* bacilli could be aerosolised following very vigorous shaking. Successful *Mtb* transmission to guinea pigs was achieved in a minority of cases but under unnatural conditions. Even with complete drying of dust particles the chances of *Mtb* transmission were low. Earlier experiments probably failed because sputum specimens were not dry enough or the air speeds were too low. Only after complete drying and intense dispersion of infected material and delivering it by bellows directly into the guinea pig’s mouth did Cornet elicit experimental infection [29]; but any remaining dampness allowed conglomeration of dust particles inhibiting aerosolization [39].

Regarding the effects of drying on coughed droplets it is also relevant to recall the correspondence between Conrad Wissemann [44] and Flügge following Flügge’s landmark 1897 paper. Wissemann pointed out that in the absence of sufficient humidity droplet components such as mucin might dry and the droplets would shrink and could then be considered as “air dust” (*Luftstäubchen);* the smaller the droplets the more rapidly drying might occur. This could also be observed when droplets settled on glass slides and fluid rapidly evaporated. Wissemann suggested that in some instances Flȕgge’s droplet infection might be more accurately considered an “air-dust” infection.

Kӧhlisch’s animal experiments provided an interesting sidelight on inhalation of microorganism infected dust particles in comparison to that of infected mucous droplets [42]. Droplets of respiratory mucous penetrated much further into bronchi and bronchioles of guinea pigs than those associated with dust particles that appeared to cause considerable inflammation in bronchial and bronchiolar mucosa inhibiting further ingress of the relevant microorganisms.

In the light of these studies it is of considerable interest that using more sophisticated methodologies and culture of coughed aerosols from sputum microscopy-positive or culture-positive PTB patients very similar results were recently recorded with negative *Mtb* culture from aerosols coughed by approximately 40% of evaluated sputum microscopy and/or *Mtb* culture positive PTB patients. This confirmed again the divergence between positive sputum microscopy for acid-fast bacilli and/or *Mtb* culture results and the coughing of microscopy-positive droplets or aerosol-positive culture material [45–48]. As already described by Flügge’s colleagues more recent studies have also found that severely ill, bedridden patients were less likely to cough viable *Mtb* containing droplets [48, 49].

It was thus not surprising that attempts to infect guinea pigs housed in small boxes coughed at by unselected phthisis patients down relatively short tubes were frequently not successful in a majority of exposed guinea pigs [30, 35]. This cast doubts upon the reliability of the methodology. Heymann reported that of 25 guinea pigs exposed to coughing of sputum-microscopy *Mtb* culture-positive patients only 6 (24%) had post-mortem evidence of TB-infection; others also recorded similar results. [33]. When Heymann took more care selecting phthisis patients for infection-transmission studies he reported a significant increase in the percentage of exposed guinea pigs becoming *Mtb*-infected [30]. He also calculated that taking into account the quantum of air breathed by a guinea pig in comparison to that of a human 100 guinea pigs might be needed to match the respiratory capacity of adult human subjects.

In 1921 a final study of *Mtb* containing droplets coughed by PTB patients down a short tube towards guinea pigs housed in boxes was reported by Hippke [50]. *Mtb*-infection transmission to guinea pigs was studied in relation to coughed droplet size and numbers of bacilli coughed and Hippke reported that stable infection confirmed by post-mortem correlated with sputum microscopy findings of much smaller coughed droplets containing relatively few bacilli. Hippke noted that the smallest droplets might measure only 15–20 μm or less on microscope slides and carry very few bacilli even only a single bacillus. Hippke also determined whether patients coughed bronchial or oral droplets finding the former more efficient for infection transmission.

The nature of droplets coughed by PTB patients also elicited differing opinions. Heymann [30] described droplets as spherical, but oval at times with three concentric layers: an acellular outer coat, an intermediate collar of cellular material and cells and a central area of slime, threads of fibrin or leukocytes and in the middle frequently bacilli alone or gathered into colonies. Variants carrying slime flakes and a light slime cover were approximately 500 μm in diameter, containing more than 200 bacilli and might have arisen directly from a TB lung-focus. Considerably smaller droplets found on the furthest microscope slides consisted mainly of slime and carried leukocytes and scattered oral epithelial cells with considerably less extraneous material, probably aiding their flight, but frequently only 2–3 bacilli. Ziesché [35] considered that a simple division of droplet types could be oral and bronchial droplets; the latter often carrying surprising numbers of TB bacilli, but infrequently other microorganisms. The addition of saliva to these droplets was typical of oral droplets that he describes as often larger size, containing oral epithelial cells and frequently microorganisms such as streptococci and staphylococci in addition to occasional *Mtb*; these sank rapidly to ground compared to bronchial droplets that had greater “propulsive force”. He thus regarded oral droplets as relatively innocuous for TB spread carrying *Mtb* bacilli relatively infrequently and sinking more rapidly to ground.

However, Koeniger [40], like Laschtschenko [34], commented that considerable numbers of bacilli might be found in oropharyngeal fluids; the numbers “freed” from tenacious mucoid sputum, might depend upon the length of time spent in the oral cavity exposed to oral fluids and enzymes. Thus tenacious mucoid bronchial sputum might be less likely associated with infection transmission than more watery oral droplets carrying fewer bacilli.

### Aseptic surgery

Stimulated by the Breslau presence of Johann von Mickulicz-Radecki, known for promoting aseptic surgery, Flügge discussed surgical wound infections with him, but doubted that contact was a likely means of transmission during surgery [1, 51]. Also air currents within a surgical theatre probably had insufficient strength to aerosolize dried sputum particles carrying microorganisms. It was more likely that droplets aerosolized by speech, coughing or sneezing contributed to surgical wound-infection; further the presence of significant numbers of spectators, frequently speaking during surgery, also multiplied wound infection risks that would be undiminished even by considerable distances from patients as infected droplets could be carried by slightest air currents; patients themselves might be a source of wound-infection. Flügge’s discussions with Mickulicz contributed to the introduction of surgical masks for all present during surgery.

## Call to Berlin and socio-economic and residential hygiene concerns

**Fig. 2:**
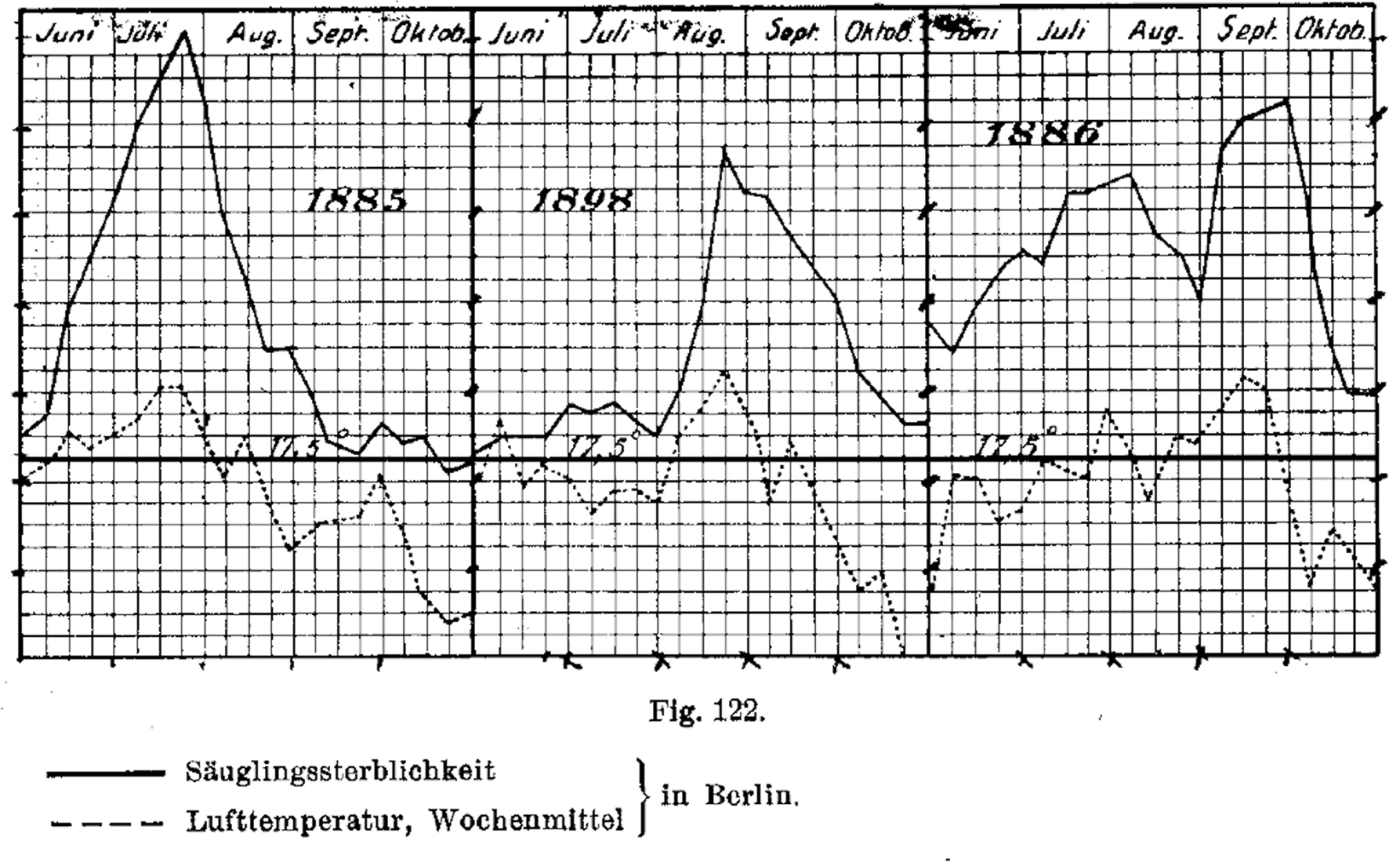
Relationship between ambient daily temperature (Lufttemperatur) and infant deaths (Sauglings sterblichkeit) in Berlin 1885, 1898 & 1886 [4]

In 1903 Flügge was offered the exceptional honour of a hygiene professorate in Vienna which he declined. In 1906 he received an honourary doctorate from Aberdeen University. Finally in 1909 at age 61-years he accepted a call to Berlin succeeding Max Rubner as head of the Berlin Hygiene Institute, the largest such institution in Germany, entering a new career phase with increased emphasis on socio-economic conditions and their influence on health and well-being. He was already well aware of the very high infant mortality in Berlin each summer as result of vomiting and diarrhoea. He records that in August 1900 and 1901 87 and 76 breast fed infants respectively died, but 1286 and 1445 artificially fed infants respectively. Also at this time approximately 66.8% of Berlin infants were artificially fed and only 33.2% breast fed [4]. He also noted the association of high summer temperatures with the occurrence of infant deaths resulting from gastro-intestinal infections (Fig. 2).

Thus it was a logical step that one of his first actions in Berlin was to create an independent Chair of Social Hygiene, but this elicited considerable resistance amongst the Berlin establishment, including Max Rubner [11].

Characteristic of this period was Flügge’s statement that “Property speculation and health do not belong together.” A major monograph “*Grosstadtwohnungen und Kleinhaussiedlungen*” (City Residences and small House Developments*)* emphasized the serious social and health damage caused by infectious disease spread and multiplication in the many barrack-like housing developments in burgeoning cities [15]. Flügge wished that “green vegetation” should be present within a 5-minute walk of all city buildings. On a positive note earlier studies in Breslau demonstrated that although temperatures might rise excessively inside crowded rooms during winter as result of over-heating there was no evidence of an accumulation of toxic chemicals produced by humans and the discomfort that individuals complained of could be easily managed by readjustment of air access and flow that eliminated symptoms of ill health [52].

## Conclusion

Carl Flügge’s career spanned a remarkable period in the development of medical science and the associated health services that initially were solely concerned with individual patients and managed by individual practitioners with little concern for broader issues, such as the environment within which various diseases occurred and the precipitating causes of disease. However, in England, similarly troubled, the Public Health Act (1848) resulted in improvements in housing and management of refuse and sewage within the burgeoning cities; these improvements in turn led to improvement in public health that was widely acknowledged [53]. Voices were also raised in Germany concerning the role of poverty and inadequate housing, nutrition and poor sanitary services in promoting disease. Johan Peter Frank was an early advocate of improved social circumstances in disease prevention and health promotion [54]. Max Pettenkofer [17] and Rudolf Virchow [16] voiced concerns regarding the epidemic occurrence of preventable communicable diseases and the impact of social conditions on nutrition and the consequences of malnutrition and inadequate housing leading to preventable infant and adult deaths.

Carl Flügge thus entered the medical field at a point when the means were rapidly becoming available to study in detail multiple microbial pathogens responsible for communicable disease and to detect specific nutritional deficiencies and do so with increasing reliability. Flügge recognized the opportunities presented to curious, concerned individuals to study various diseases and promote the means to prevent and manage these and so alleviate human suffering even in the absence of immediate socio-economic improvements.

Flügge was never content with merely describing the many organisms causing human disease but was always concerned to identify the underlying causes of disease, its spread and the life cycles of relevant organisms and prevention of infection and precise details of the living circumstances of those infected. Having identified the aerogenous transmission of *Mtb* by coughed infected droplets his colleagues Heymann [31] and Bartenstein [55] on Flügge’s instigation, showed that the simple means of holding a handkerchief or hand 5 cm in front of a cough indirectly, but markedly, reduced the incidence of *M tuberculosis* infections in experimental animals. This expedient would likely be even more effective were the handkerchief held much closer to the mouth. Following on recent experience of the COVID-19 pandemic it is likely that the wider adoption of Flügge’s suggestion to “shelter one’s coughs” as a socially important response to coughing would not only reduce TB-transmission but also transmission of other respiratory infections such as COVID-19.

It was also typical of Flügge’s character that the acrimonious academic debate regarding whether TB infection was the result of the inhalation of dried sputum fragments, as supported by Koch [28] and Cornet [29] or recently coughed droplets as propounded by Flügge and his colleagues, never influenced his close personal relationship with Robert Koch. On the occasion of Koch’s 60th birthday Flügge contributed a paper to a celebratory “Festschrift”. This paper “*Untersuchungen über die hygienischen Bedeutung einiger klimatischer Faktoren*,* insbesonderere des Windes”* (Investigation of the hygiene implications of certain climatic factors and in particular the winds) commences with reference to Koch as his “friend and mentor” [56]. Koch for his part thanked Flügge for accepting him as his student referring to Flügge’s provision of hygiene material for his lectures following Koch’s appointment to the Berlin Chair of Hygiene in 1885 [24].

Flügge’s monumental work “*Grundriss der Hygiene*” in its 7th edition (1912) runs to 847 pages encompassing detailed descriptions of every aspect of an ideal environment in which human life might best survive and flourish [4]. It is thus noteworthy in the light of Flügge’s career and his emphasis on the environment and its close relationship with health that in 1972, nearly 50 years after Flügge’s death, the interdependent relationship between the environment, health and human rights was finally recognized in a declaration by a United Nations Conference in Stockholm on the Human Environment (Stockholm Declaration). While the right to health was recognized in the Universal Declaration of Human Rights in 1948 and the International Covenant on Economic, Social and Cultural Rights in 1966, it was only in 2022 that the United Nations General Assembly resolution 76/300 also recognized the human right to a clean, healthy and sustainable environment [57].

On the 10th anniversary of Flügge’s death Walther Liese, an earlier student and assistant of Flügge in Berlin, reflected on meeting Flügge shortly before his death [58]. Flügge remarked that the age of classical experimental hygiene had passed its peak. Future research belonged to those with technical knowledge of the many functions of hygiene in all its aspects *(Gesundheitstechnik*). Looking back from the vantage point of the century elapsed since Flügge’s death it is clear that already in 1923, it would have been almost impossible for any single individual to amass the newly developing technical and analytical skills covering the wide range of subjects that Flügge studied, researched and wrote about during his long career. Carl Flügge was indeed the last of the great holistic hygienists. At the same time, taking into account the many different facets of his career, he could also be counted as one of the first campaigners for the human right of appropriate, universal, environmental health in all its aspects [11].

## Data Availability

No datasets were generated or analysed during the current study.
